# Orbital metastasis secondary to pulmonary adenocarcinoma treated with gefitinib: a case report

**DOI:** 10.1186/1752-1947-6-353

**Published:** 2012-10-18

**Authors:** Yasuko Koma, Keiko Goto, Chihiro Yoshida, Kengo Kimura, Yusuke Matsumoto, Midori Koyama, Nariyasu Nakashima, Daiki Masuya, Hirofumi Matsuoka, Harukazu Yoshimatsu, Atsushi Azumi, Yujiro Suzuki

**Affiliations:** 1 Respiratory Center, Shinko Hospital, 1-4-47, Wakinohamacho, Chuo-ku, Kobe 651-0072, Japan; 2 Department of Ophthalmology, Kobe Kaisei Hospital, 3-11-15, Shinoharakitamachi, Nada-ku, Kobe 657-0068, Japan

## Abstract

**Introduction:**

Orbital metastases of lung cancer are rare. However, because the number of patients diagnosed with lung cancer is increasing, the probability that a physician will see a patient with an orbital metastasis is also increasing. Unfortunately, the clinical course and response of these patients to cytotoxic chemotherapy are generally poor and keeping a patient’s quality of vision is difficult. In recent years, gefitinib, an epidermal growth factor receptor tyrosine kinase inhibitor, has brightened the outlook for patients with advanced non-small cell lung cancer, especially for those who carry epidermal growth factor receptor-activating mutations.

**Case presentation:**

A 62-year-old Japanese man presented with swelling of the eyelid margin and ptosis of his right eye. A physical examination revealed double vision in his right eye and an alteration in elevator muscle mobility. A magnetic resonance image demonstrated a right intra-orbital mass (18 × 16mm). Screening examinations were carried out because this mass was suspected to be a metastasis from another organ. Chest computed tomography revealed a 42 × 37mm mass shadow on the left side of the hilum with mediastinal lymph node metastases. Adenocarcinoma with an epidermal growth factor receptor gene mutation (exon 19 deletion L747-E749; A750P) was detected in a transbronchial biopsy specimen; the patient was diagnosed with stage IV (T2N2M1) non-small cell lung cancer.

Gefitinib (250mg/day) was chosen as first-line chemotherapy because there was no pre-existing interstitial shadow. After two months of treatment, the patient’s right eye opened completely and follow-up magnetic resonance imaging revealed a marked reduction of the intra-orbital mass to 14 × 13mm. Three months after treatment initiation, a follow-up computed tomography showed a marked reduction in the size of the primary lesion to 23 × 20mm. The patient is continuing gefitinib treatment without any adverse effects noted on computed tomography, physical, or laboratory examination.

**Conclusions:**

We report the case of a patient with an orbital non-small cell lung cancer metastasis with epidermal growth factor receptor-activating mutations. This metastasis, as well as the primary lesion, showed a marked response to the molecular targeting drug gefitinib, and the patient’s vision was kept without an invasive procedure. Gefitinib may be a good first choice for patients with orbital non-small cell lung cancer metastasis harboring epidermal growth factor receptor-activating mutations.

## Introduction

Orbital metastases of cancer are rare, accounting for 7% of intra-orbital tumors [1]. They most commonly originate from primary lesions of the breast (48%), prostate and skin (melanoma) (12%), lung (8%), and kidney (7%) [1]. The clinical course of patients with orbital metastases of lung cancer depends on the nature of the primary tumor, such as its histological types; the prognosis is poor in most cases with an average survival of 7.4 months [2].

However, recent studies have revealed the favorable efficacy of gefitinib, an epidermal growth factor receptor (EGFR) tyrosine kinase inhibitor, compared with that of combination cytotoxic chemotherapy in patients with EGFR-activating mutations [3]. To the best of our knowledge, no report has published the effect of gefitinib on an orbital metastasis.

Here we present the case of a patient with orbital metastasis of lung adenocarcinoma which showed a good response to gefitinib therapy in both the primary lesion and orbital metastasis improving the patient’s quality of vision.

## Case presentation

A 62-year-old Japanese man presented with swelling of the eyelid margin and ptosis of his right eye. He was a heavy cigarette smoker (30 cigarettes per day for 50 years). He had no other significant medical history. A physical examination revealed double vision in the right eye and an alteration in elevator muscle mobility. The patient’s visual acuity was not reduced; there was no increased intra-ocular pressure.

**Figure 1:**
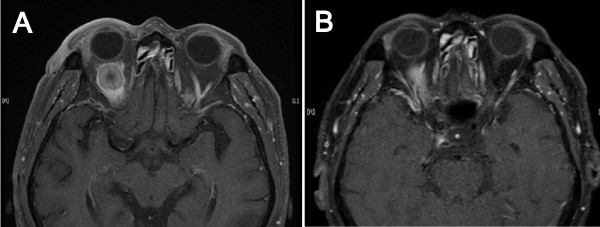
**Brain magnetic resonance image.A**: Showing an intra-orbital mass on the right side before gefitinib therapy. **B**: Revealing a reduction of the intra-orbital mass after two months of gefitinib therapy

**Figure 2:**
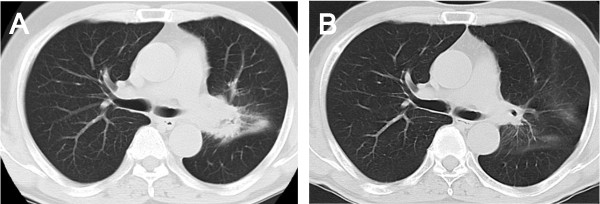
**Chest computed tomography images.A**: Showing a mass shadow on the left side of the hilum before gefitinib therapy. **B**: Revealing a good response of the primary tumor after three months of gefitinib therapy

A magnetic resonance image (MRI) demonstrated a right intra-orbital mass (18 × 16mm; Figure 1A) with bone destruction at the left temporal bone (figure not shown). Screening examinations were carried out because this mass was suspected to be a metastasis from another organ. Chest computed tomography (CT) revealed a 42 × 37mm mass shadow on the left side of the hilum with mediastinal lymph node metastases (Figure 2A). Adenocarcinoma with an EGFR gene mutation (exon 19 deletion L747-E749; A750P) was detected in a transbronchial biopsy specimen; the patient was diagnosed with stage IV (cT2N2M1b) non-small cell lung cancer (NSCLC). The tumor marker carcinoembryonic antigen (CEA) was elevated to 71.2ng/mL (normal range <5ng/mL).

Gefitinib (250mg/day) was chosen as the first-line chemotherapy because there was no pre-existing interstitial shadow. The patient’s ptosis slightly improved after two weeks of therapy; after two months of treatment his right eye opened completely and exhibited improved mobility. Follow-up MRI revealed a marked reduction of the intra-orbital mass to 14 × 13mm (Figure 1B). Three months after treatment initiation, a follow-up CT showed a marked reduction in the size of the primary lesion to 23 × 20mm (Figure 2B). In addition, CEA decreased to 5.5ng/mL. The patient is continuing gefitinib treatment without any adverse effects noted on CT, physical, or laboratory examination.

## Discussion

Orbital metastases have been reported to originate in many organs. No matter from where the primary lesion originates, orbital metastases generally lead to an alteration in ocular motility, and proptosis due to an increase in intra-orbital volume that reduces vision [4]. Of cases with orbital metastases and tumor invasion of the optic nerve, 20% showed a reduction in vision [5].

Because treating an orbital metastasis is not a complete cure, therapy targeting the primary lung cancer must take precedence. Sometimes an invasive procedure, radiotherapy and orbital surgery may be undertaken to relieve the patient’s pain or because the patient’s vision is important to their quality of life [1].

The number of patients diagnosed with lung cancer is increasing and because the lung is one of the most frequent reported origins of orbital metastases, the probability that a physician will see a patient with an orbital metastasis is also increasing. The most frequent pathological types are adenocarcinoma (65%) and more than half of them are of the poorly differentiated type [2]. The EGFR mutation rate of cases with orbital metastases is unknown.

The choice of a systemic therapy agent for stage IV lung cancer with metastases depends on the histological type of the primary tumor. In small cell lung cancer (SCLC), cytotoxic chemotherapy (usually a platinum-based combination therapy) is effective for orbital metastases as well as for the primary lesion. Unfortunately, cytotoxic chemotherapy does not show as quick a response for NSCLC as it does for SCLC. Recently, several novel antitumor agents have brightened the outlook for patients with advanced NSCLC; in fact, Zarogoulidis *et al.* reported favorable efficacy of combination chemotherapy including bevacizumab and pemetrexed in patients with orbital metastasis [6]. Gefitinib shows a rapid and improved response rate over combination chemotherapy in patients with NSCLC harboring EGFR-activating mutations [3]. The efficacy of gefitinib could be expected to apply not only to the primary lesion, but also to metastatic lesions. This case showed the efficacy of gefitinib to orbital metastasis with high response rate. Thus, the patient’s quality of vision could be saved without an invasive procedure. Unfortunately, however, patients usually acquire resistance to gefitinib around 12 months of treatment [3]. Further studies will be needed to elucidate the efficacy of gefitinib on metastasis.

## Conclusion

Here we present the case of a patient with orbital metastasis of lung adenocarcinoma in which a marked positive response to gefitinib therapy was shown in both the primary lesion and orbital metastasis. Gefitinib therapy improved the patient’s quality of vision without an invasive procedure. Patients with orbital metastases are in need of highly effective treatment if their vision is to be spared; gefitinib may be a good first choice for patients with NSCLC and EGFR-activating mutations.

## Consent

Written informed consent was obtained from the patient for publication of this case report and accompanying images. A copy of the written consent is available for review by the Editor-in-Chief of this journal.

## Competing interests

The authors declare that they have no competing interests.

## Authors’ contributions

YK analyzed and interpreted the patient data, and was a major contributor in writing the manuscript. KG analyzed and interpreted the patient data regarding pulmonary oncology. CY, KK, YM, MK, NN, DM, HM and HY performed the bronchoscopic examination. AA performed the physical examination of the eye and interpreted the patient data regarding ophthalmological disease. YS supervised the concept and design of the manuscript. All authors read and approved the final manuscript.
